# Cancer Vaccine Therapeutics: Limitations and Effectiveness—A Literature Review

**DOI:** 10.3390/cells12172159

**Published:** 2023-08-28

**Authors:** Mariusz Kaczmarek, Justyna Poznańska, Filip Fechner, Natasza Michalska, Sara Paszkowska, Adrianna Napierała, Andrzej Mackiewicz

**Affiliations:** 1 Department of Medical Biotechnology, Poznan University of Medical Sciences, 61-866 Poznań, Poland; 2 Department of Cancer Diagnostics and Immunology, Greater Poland Cancer Center, 61-866 Poznań, Poland; 3 Scientific Society of Cancer Immunology, Poznań University of Medical Sciences, 61-866 Poznań, Poland; justynapoznanski@yahoo.com (J.P.);

**Keywords:** tumor microenvironment, cancer vaccine, immunotherapy, cell-based, dendritic cell, whole-cell, viral-based, bacterial-based, peptide vaccine, genetic vaccine

## Abstract

In recent years, there has been a surge of interest in tumor microenvironment-associated cancer vaccine therapies. These innovative treatments aim to activate and enhance the body’s natural immune response against cancer cells by utilizing specific antigens present in the tumor microenvironment. The goal is to achieve a complete clinical response, where all measurable cancer cells are either eliminated or greatly reduced in size. With their potential to revolutionize cancer treatment, these therapies represent a promising avenue for researchers and clinicians alike. Despite over 100 years of research, the success of therapeutic cancer vaccines has been variable, particularly in advanced cancer patients, with various limitations, including the heterogeneity of the tumor microenvironment, the presence of immunosuppressive cells, and the potential for tumor escape mechanisms. Additionally, the effectiveness of these therapies may be limited by the variability of the patient’s immune system response and the difficulty in identifying appropriate antigens for each patient. Despite these challenges, tumor microenvironment-targeted vaccine cancer therapies have shown promising results in preclinical and clinical studies and have the potential to become a valuable addition to current cancer treatment and “curative” options. While chemotherapeutic and monoclonal antibody treatments remain popular, ongoing research is needed to optimize the design and delivery of these therapies and to identify biomarkers that can predict response and guide patient selection. This comprehensive review explores the mechanisms of cancer vaccines, various delivery methods, and the role of adjuvants in improving treatment outcomes. It also discusses the historical background of cancer vaccine research and examines the current state of major cancer vaccination immunotherapies. Furthermore, the limitations and effectiveness of each vaccine type are analyzed, providing insights into the future of cancer vaccine development.

## 1. Introduction

Tumor microenvironments (TMEs) involve a mixed composition of transforming immune cells, blood vessels, stromal cells, and extracellular matrix, creating tumors that are exclusive to their location and diverse in composition among individual patients [1]. The metastatic progression, along with the distinct and peculiar composition of the TME, plays the most significant role in the patient’s response to treatment [2] All components of the transforming TME determine the changes and behavior of surrounding macromolecules and tissue development, creating complicated variations in immune response and tumor behavior, like suppression or stimulation of tumor growth [1,3].

The first standardized cancer immunotherapy utilized for the treatment of malignant tumors was developed by William B. Coley in 1891. For 40 years following this discovery, the ‘Coley Toxin’, developed from Streptococcal bacteria, stimulated immune system responses and was used to treat patients with bone and soft tissue sarcomas, shrinking the tumor significantly. Since the end of Coley’s research in 1933, the study of TMEs in correlation to cancer vaccine development skyrocketed, leading to cures and positive treatment outcomes of liver cancer related to hepatitis B, cervical cancer associated with human papillomavirus, colon cancer, melanomas, and bladder cancer, to name a few [4].

Cancer vaccination, also referred to as cancer immunization or cancer immunotherapy, is a therapeutic approach aimed at activating the immune system to recognize and combat cancer cells. Its primary objective is to prevent tumor growth, recurrence, or metastasis while enhancing the immune system’s capacity to identify and eliminate cancer cells. The mechanisms of cancer vaccines involve eliciting an immune response targeting specific tumor-associated antigens (TAAs), which are proteins expressed by cancer cells. This immune response involves the activation of T cells, B cells, and other immune cells, leading to the destruction of cancer cells. Cancer vaccines can serve as preventive measures in high-risk populations, known as prophylactic vaccines, and as treatment options for individuals already diagnosed with cancer, referred to as therapeutic vaccines. By harnessing the power of the immune system, cancer vaccination holds promise in providing effective strategies for cancer prevention and treatment.

Additionally, adjuvants are essential components of cancer vaccines, as they enhance immune responses by activating innate immune pathways. Adjuvants, such as Toll-like receptor (TLR) agonists, cytokines, and immune checkpoint inhibitors, have been utilized to improve vaccine efficacy. TLR agonists, such as CpG oligodeoxynucleotides (CpG-ODNs), stimulate antigen-presenting cells (APCs) and promote antigen presentation, while immune checkpoint inhibitors block inhibitory signaling pathways, allowing sustained immune activation. These adjuvants have shown promising results in preclinical and clinical studies, contributing to the development of novel cancer vaccines. Despite the challenges of cancer vaccine development regarding efficacy, several therapeutic vaccination strategies are under development and are being evaluated in preclinical and clinical trials [5].

Despite the progress made in the field of cancer vaccines, it is important to address the limitations and potential side effects of cancer vaccines. Tumor heterogeneity, immunosuppressive TMEs, and immune tolerance mechanisms pose significant challenges for vaccine efficacy. The identification of suitable TAAs and the selection of optimal adjuvants remain critical for successful vaccine development, and the overall effectiveness of cancer vaccines may vary among different cancer types and individual patients, necessitating personalized approaches. Common side effects include injection site pain, headache, influenza-like illness, fever, nausea, diarrhea, rashes, erythema, pruritus, myalgia, and dyspnea. Serious adverse events are less common but may involve immune system disorders, psychiatric disorders, and pulmonary embolism. While rare, varying levels of toxicity have been observed in some cases. Vaccines and their adjuvants can also lead to additional complications, such as hyponatremia, liver enzyme elevation, anemia, colitis, and increased creatinine levels. Furthermore, vaccine-induced immune responses, particularly T cell responses, have the potential to cause tumor pseudo-progression, as highlighted by M. Platten et al. (2021) [6]. It is essential to carefully monitor and manage these immune-related reactions to ensure the safety and efficacy of cancer vaccines.

In this comprehensive review article, we provide a concise overview mechanism underlying cancer vaccines, the various delivery methods employed, and the role adjuvants have in enhancing treatment outcomes. We also delve into the historical background of cancer vaccine research and explore the current state of four major cancer vaccination immunotherapies, including traditional cell-based vaccines, second-generation microbial vector vaccines, peptide vaccines, and third-generation genetic vaccines. Furthermore, we discuss the limitations and effectiveness of each vaccine type to provide a well-rounded perspective on the future of cancer vaccine development.

## 2. Cellular Composition of Tumor Microenvironment

Cancer vaccine therapeutics hold great promise in stimulating the immune system to target cancer cells. However, their effectiveness is influenced by the complex cellular composition of the TME. The TME encompasses immune cells such as T cells, B cells, and natural killer cells (NK), each playing distinct roles in the anti-tumor immune response. T cells exhibit phenotypic plasticity, allowing them to differentiate into effector T cells or immunosuppressive regulatory T cells (Tregs) [3,7]. Immune checkpoint receptors expressed by T cells, such as PD-1 and CTLA-4, modulate T cell activation and function. Blocking these immune checkpoint pathways has shown success in cancer immunotherapy.

B cells, on the other hand, possess multifaceted functions within the TME. They can produce antibodies, regulate antigen processing and presentation, and exhibit pro- and anti-tumorigenic properties [8]. Regulatory B cells (Bregs) secrete immunosuppressive proteins, like IL-10 and IL-35, dampening the anti-tumor immune response [1,9]. The dynamic interactions between these immune cells and the tumor cells within the TME shape the efficacy of cancer vaccines. Furthermore, the TME comprises other components, such as dendritic cells (DCs), neutrophils, tumor-associated macrophages (TAMs), and cancer-associated fibroblasts (CAFs), each with distinct contributions to tumor progression and immune modulation.

While cancer vaccines hold the potential to stimulate the immune system to target cancer cells, there are limitations to their effectiveness. The TME’s immunosuppressive nature, genetic instability, and heterogeneity pose challenges to cancer vaccine therapy. Targeting specific components of the TME, such as immune checkpoints or fibroblast activation, may help overcome these limitations and enhance the effectiveness of cancer vaccines.

## 3. Mechanisms of Cancer Vaccines

Cancer vaccines employ various mechanisms to stimulate the immune system and generate an effective anti-tumor response. One common approach involves the use of DCs, which are potent APCs. In one method, DCs are collected from the patient’s blood or generated in the laboratory. They are then matured and activated using immune-stimulating molecules or tumor antigens. After loading the DCs with tumor-specific antigens (TSAs) derived from tumor cells or genetic material, the loaded DCs are administered back to the patient. These DCs migrate to lymphoid organs, where they interact with immune cells, such as T cells, B cells, and NK) cells. The DCs present the tumor antigens to CD4+ helper T cells and CD8+ cytotoxic T lymphocytes (CTLs), leading to their activation. The activated T cells provide help signals to other immune cells, enhancing the immune response against tumor cells. CTLs specifically recognize and target cancer cells expressing the tumor antigens, resulting in their elimination. Additionally, this vaccine aims to induce a memory response, allowing for a more effective immune response upon subsequent encounters with tumor cells.

Another strategy involves the use of whole-cell preparations derived from cancer cells. Cancer cells are collected from the patient’s tumor or established cancer cell lines. These cells are inactivated or genetically modified to reduce their ability to grow and cause disease. When administered back to the patient, the whole cells are recognized by various immune cells, including DCs, macrophages, and NK cells, triggering an immediate non-specific inflammatory response. The activated immune cells, in turn, process the tumor antigens, present them to T cells, and initiate an immune response. CD4+ helper T cells provide help signals to other immune cells, while CD8+ CTLs recognize and eliminate tumor cells expressing the presented antigens. Whole-cell cancer vaccines also aim to induce a memory response for enhanced immune protection against tumor recurrence.

Induced pluripotent stem cell (iPSC)-based cancer vaccines represent a promising approach. iPSCs are generated from somatic cells and differentiated into TME-specific cells, such as TAFs, endothelial cells, or immune cells. These iPSC-derived cells express antigens characteristic of the TME, including TSAs or molecules involved in immunosuppression. Upon administration to the patient, these cells are recognized by immune cells, leading to the activation of a robust immune response. APCs, primarily DCs, take up iPSC-derived antigens, process them, and present them to T cells. The activated T cells, particularly CD8+ CTLs, recognize and target TME components expressing TSAs. The expansion of effector cells contributes to the elimination of tumor cells, and the induction of a memory response enables a more rapid and effective immune response upon subsequent encounters with tumor cells.

In situ cancer vaccines are administered directly into the tumor site or a nearby lymph node. This approach involves the activation of APCs, such as DCs, macrophages, neutrophils, and NK cells, within the TME. The vaccine induces an inflammatory response, cytokine production, and immune cell recruitment. APC take up tumor antigens released during vaccine administration, process them, and present them to T cells. This triggers the activation of CD8+ CTLs and CD4+ helper T cells, which work synergistically to eliminate tumor cells. The activated immune cells produce effector molecules and mediate the destruction of tumor cells within the TME. In situ cancer vaccines also aim to generate a memory response for heightened protection against tumor recurrence.

Viral-based cancer vaccines utilize modified viruses to directly activate the immune response. The modified virus interacts with immune cells, including DCs, macrophages, and NK cells, triggering an inflammatory response and the release of pro-inflammatory cytokines and chemokines. The virus particles are phagocytosed by immune cells, and the TAAs expressed by the virus or delivered to infected cells are processed and presented to T cells. CD8+ CTLs recognize the presented TAAs, leading to their activation and expansion. CD4+ helper T cells provide help signals to other immune cells, and antibodies produced against the TAAs can directly bind to tumor cells, facilitating their destruction. Viral-based vaccines aim to induce a memory response, enabling a more rapid and robust immune response upon subsequent encounters with tumor cells expressing the same TAAs.

Similarly, bacteria-based cancer vaccines utilize modified bacteria to activate the immune response. The modified bacteria interact with immune cells, triggering an inflammatory response and the production of pro-inflammatory cytokines, chemokines, and other signaling molecules. APCs, particularly DCs, phagocytose the bacteria and process them, leading to the presentation of TAAs. CD8+ CTLs recognize the presented TAAs and become activated, while CD4+ helper T cells provide help signals to other immune cells. Antibodies produced against the TAAs can directly bind to tumor cells, facilitating their destruction. Bacteria-based vaccines aim to induce a memory response, leading to a more rapid and robust immune response upon subsequent encounters with tumor cells expressing the same TAAs.

Peptide cancer vaccines involve the administration of specific peptides derived from TAAs. These peptides are taken up by APCs primarily DCs, which process them and present them on their surface using major histocompatibility complex (MHC) molecules. CD8+ CTLs recognize the presented peptides on MHC class I molecules, leading to their activation and expansion. CD4+ helper T cells recognize the peptides presented on MHC class II molecules and provide help signals to other immune cells. B cells can be activated by the peptides presented by DCs, leading to the production of antibodies specific to the TAAs. The antibodies can directly bind to tumor cells, facilitating their destruction, and a memory response is induced for enhanced immune protection.

DNA and RNA cancer vaccines involve the administration of DNA or RNA molecules encoding TAAs. The administered DNA or RNA is taken up by cells, such as muscle cells or DCs, and the TAAs are produced within these cells. APCs cells, particularly DCs, take up the TAAs and present them on their surface using MHC molecules. CD8+ CTLs recognize the presented TAAs on MHC class I molecules, leading to their activation and expansion. CD4+ helper T cells recognize the TAAs presented on MHC class II molecules and provide help signals to other immune cells. B cells can be activated by the TAAs presented by DCs, leading to the production of antibodies specific to the TAAs. The antibodies can directly bind to tumor cells, facilitating their destruction, and a memory response is induced for enhanced immune protection.

Exosome-based cancer vaccines utilize exosomes loaded with TAAs or nucleic acids encoding TAAs. These exosomes are taken up by APCs cells, primarily DCs, which become activated and enhance their antigen-presenting capabilities. The TAAs delivered by exosomes are processed and presented on the surface of DCs using molecules. CD8+ CTLs recognize the presented TAAs on MHC class I molecules, leading to their activation and expansion. CD4+ helper T cells recognize the TAAs presented on MHC class II molecules and provide help signals to other immune cells. B cells can be activated by the TAAs presented by DCs, leading to the production of antibodies specific to the TAAs. The antibodies can directly bind to tumor cells, facilitating their destruction, and a memory response is induced for heightened immune protection.

## 4. Cancer Vaccine Progress and Development

Tumor cells are known to exhibit genetic instability, resulting in numerous somatic mutations, such as deletions, insertions, point mutations, and translocations. This genetic complexity can lead to the production of abnormal proteins, making them attractive targets for immunotherapy. By focusing on patient-specific proteins, immunotherapy offers a potential solution to address challenges associated with self-tolerance and treatment efficacy [10].

The average age of cancer diagnosis is around 66 years, and as patients age, the TME becomes increasingly complex. Consequently, it is imperative to explore alternative targets for cancer vaccines that specifically address age-related variations in the TME, thereby optimizing treatment outcomes [11]. In a study by Grizzle et al. (2007), it was observed that older mice had an increased presence of myeloid-derived suppressor cells in the TME compared to younger mice, resulting in impaired T cell responses [12]. This highlights the decline in efficacy and dysregulation of the immune system, necessitating the use of adjuvants and strategies to elicit robust cellular responses in cancer vaccines [13].

Compared to conventional treatment methods, such as chemotherapy and radiotherapy, vaccine immunotherapy has emerged as a powerful tool and an area of intense research for inducing or reactivating anti-tumor immune responses [14]. Recent advancements have revealed the potential of vaccine immunotherapy to precisely target specific tumors within specific regions of the body, enabling tailored interventions at the cellular and tissue level. These breakthroughs represent considerable progress in cancer treatment strategies, offering promising outcomes on a global scale.

The selection of a cancer vaccine is a critical decision that can impact the speed, intensity, and duration of the immune response. The clinical effectiveness of a given treatment is determined by its ability to prolong patient survival, improve clinical response, achieve partial response, or enhance disease stability. Several factors, including cancer stage, patient age, the immunosuppressive nature of the TME, and the choice of antigens and adjuvants, influence treatment outcomes [13,15]. The variability of treatment responses among different population cohorts can be substantial, with exceptional success observed in some cases but limited efficacy in specific subgroups [16]. Despite the promising potential of vaccine immunotherapy, achieving consistently positive clinical outcomes remains a significant challenge, which has raised concerns about its widespread acceptance [16].

Cancer vaccines are meticulously developed and tailored to target specific antigens, with the aim of modulating the immunosuppressive TME and eliciting effective immune responses. A key focus of research lies in combining cancer vaccines with various therapies, such as radiotherapy, oncolytic viruses, cytokine-based approaches, and physical therapy, to enhance their efficacy and impact [17]. Vaccines can be designed for TAAs, TSAs, cancer germline antigens (CGAs), and virus-associated antigens, each serving unique roles in cancer immunotherapy [18].

TAAs are expressed in minimal amounts in healthy cells but are overexpressed in tumor cells, often because of posttranslational modifications. Despite extensive research on TAAs, such as prostatic acid phosphatase for prostate cancer and carcinoembryonic antigen for gastrointestinal cancer, their limited tumor-specificity has hindered their success as therapy targets. Notably, chimeric TAA receptor T cell therapy targeting CD19 has shown promise in patients with acute lymphoblastic leukemia [19]. However, it is important to consider that targeting TAAs can lead to adverse effects, including colitis, hepatitis, and in some cases, even death [20].

In contrast, TSAs are absent in healthy cells and arise from nonsynonymous mutations. Since TSAs are typically absent from normal tissue, targeting them in therapy offers the potential to minimize the risk of autoimmune responses, making them a promising target for cancer treatment [18,21]. Unlike TAAs, TSAs are not subjected to central tolerance mechanisms that eliminate T and B cells reactive to self-antigens.

CGAs, on the other hand, are predominantly expressed in reproductive tissues, such as trophoblast and fetal ovaries. They are selectively expressed in specific tumor types due to epigenetic dysregulation. Virus-associated antigens originate from oncogenic viral proteins that integrate into the host genome, promoting tumorigenesis [18]. All these mentioned antigens can be presented by the major histocompatibility complex and recognized by T cells, playing crucial roles in immunotherapeutic approaches against cancer.

The main cancer vaccine types discussed in this review are based on composition and include (1) tumor or immune cell-based vaccines, (2) peptide-based vaccines, (3) microbial vector-based vaccines, (4) nucleic acid-based vaccines, (5) exosome-based vaccines, (6) induced pluripotent stem cell-based vaccines, (7) in situ vaccines, and finally (8) a discussion on combination vaccine methods. Table 1 provides a comprehensive overview of the mechanisms underlying each vaccine, detailing the specific stimuli employed, the elicited T cell responses, and the accessory cells involved in the process (Table 1).

### 4.1. Cell-Based Vaccines

Cell-based vaccines use immune cells, such as DCs, to stimulate an immune response against cancer cells. The mechanism of action of cell-based vaccines involves the collection of immune cells from a patient’s blood or tumor and the activation and expansion of these cells in a laboratory setting. The activated immune cells are then reintroduced into the patient’s body, where they target and attack cancer cells.

Cell-based vaccines offer two approaches: allogeneic and autologous tumor cells. Allogeneic vaccines, while lacking personalization, present a time-saving advantage. In contrast, autologous vaccines utilize the patient’s own tumor cells, ensuring antigen compatibility but at the expense of increased costs and time requirements. The selection between these approaches depends on several factors, including the patient’s specific needs, cancer stage, and resource availability.

One significant drawback of cell-based vaccines is the potential for human leukocyte antigen (HLA) mismatch. This mismatch can shift the focus onto HLA molecules rather than the immune system itself, affecting the effectiveness of the vaccine [31]. Cell-based vaccines can be further categorized into two types: DC vaccines and whole-cell vaccines.

Refining the balance between personalization and practicality is a crucial consideration in the development of cell-based cancer vaccines, as it ensures the optimal utilization of resources while maximizing the potential benefits for patients.

#### 4.1.1. Dendritic Cell Vaccines

DC vaccination, a pioneering approach explored in clinical trials during the 1990s, seeks to trigger an effective anti-tumor immune response against tumor antigens through multiple mechanisms. The fundamental principle behind DC vaccines involves introducing specific tumor antigens to the immune system, thereby activating immune cells, especially T cells. This activation empowers T cells to recognize and eliminate cancerous cells, fostering a targeted attack against the disease. Notably, DC vaccines possess the ability to induce the production of cytokines, which play a crucial role in modifying the TME. These cytokines can bolster the immune response against cancer cells and promote the growth of blood vessels, facilitating the improved delivery of immune cells and anticancer drugs to the TME. This multifaceted approach holds the potential to enhance the overall efficacy of cancer immunotherapy [32]. Despite ongoing clinical trials investigating various protocols and treatment regimens, DC vaccines have yet to realize their full potential in clinical practice [33]. Continued research and refinement of DC vaccination strategies aim to overcome existing limitations and unleash the full therapeutic power of this promising approach in the fight against cancer.

Using a cytokine maturation cocktail, DC vaccines are reduced by isolating autologous monocytes from a patient’s peripheral blood and are then exposed to cytokines initiating the differentiation of immature DCs [34]. This cytokine mixture prepares the DCs for specific functions like lymph node homing. They are then loaded with whole tumor lysate, DC-tumor cell fusion, virus, specific shared TAAs, like peptides and nucleic acid, or unique neoantigens from the tumor cells [22]. It is introduced to the patient’s immune system, causing the production and presentation of tumor antigens to CD8+ and CD4+ T cells, engaging both the innate and adaptive immunities and leading to the development of immunological memory in the case of tumor relapse [23]. Some studies reported that antigen-loaded DC vaccines induced stronger immune responses than vaccines composed of antigens and adjuvants alone [35].

Patients with late-stage disease have dominant immunosuppressive mechanisms; they can have weakened responses to DC vaccines and T cell activation leading to decreased efficacy in patients with metastasis [22]. While this concept is not entirely understood, given that most vaccines can induce an immune response against a specific antigen, the reason could pertain to the immunosuppressive TME where tumor-specific T cells, which would otherwise be inactivated to continue to expand and carry out effective functions, would then transfer the TME from suppressive to inflammatory. Duraiswamy et al. (2013) confirmed in preclinical studies that combination therapy of the tumor cell vaccine along with a blockade of programmed cell death protein 1 (PD-1) and/or cytotoxic T-lymphocyte-associated antigen 4 (CTLA-4) could improve tumor control [36].

The limited efficacy observed in DC vaccines can be attributed to suboptimal protocols that fail to generate an optimal T cell response. One factor contributing to this limitation is the use of granulocyte-macrophage colony stimulating factor (GM-CSF) for maturing peripheral blood mononuclear cells, which results in the production of monocyte-derived DCs that have a limited ability to migrate to lymph nodes [37,38]. The migration of DCs to lymph nodes is crucial for T cell-antigen interaction, and the use of monocyte-derived DC vaccines hampers this essential step [39,40,41].

Furthermore, it has been highlighted by Roy et al. (2020) that the production of DC vaccines can be costly and complex, further posing challenges to their widespread adoption [22]. These factors have contributed to the need for refinement in the manufacturing and administration of DC vaccines to enhance their effectiveness.

#### 4.1.2. Whole-Cell Vaccines

Whole-cell cancer vaccines have a rich history of development dating back to the 1950s. Among these, Bacillus of Calmette-Guérin (BCG) stands as a significant milestone. In 1990, BCG became the first approved whole-cell vaccine for cancer therapy, specifically for the treatment of bladder cancer. TICE^®^ BCG, an attenuated, live culture preparation of the BCG strain of Mycobacterium Bovis, gained United States Food and Drug Administration approval in 1998. It was intended for intravesical use to combat recurrent tumors in patients with carcinoma in situ of the urinary bladder and prevent the recurrence of Stage TaT1 papillary tumors with an elevated risk of relapse. This pioneering approval marked a significant advancement in whole-cell cancer vaccine development [42].

Whole-cell cancer vaccines are designed to trigger an immune response against multiple antigens expressed by cancer cells. Unlike targeted therapies that focus on specific antigens, these vaccines expose the immune system to the entirety of the cancer cell. By targeting the entire tumor cell, these vaccines have the potential to activate a diverse array of immune cells, including T cells, B cells, and NK cells. This comprehensive immune activation holds great promise for bolstering the immune system’s capacity to effectively target and eliminate the complex TME.

Autologous whole-cell vaccines are produced from tumor cells isolated directly from the patient, which are collected with the abundance of TAAs in the TME, making this the ideal strategy for creating a natural mode of immune response by the adaptive immune system. However, multiple inhibitory signals on T cells and the APCs responsible for activating the T cell response prevent effective immune activation and recognition of growing tumors in patients [31]. GVAX is a cancer vaccine genetically modified to secrete the immune stimulatory cytokine and GM-CSF preventing further cell division, effective immune activation, and recognition of growing tumors in patients [31,43]. One of the main concerns discussed is the prohibitive cost and complexity of their production [44].

In a notable study by Xia et al. (2016) conducted at the Center for Cancer Research, potential biomarkers were identified to distinguish productive and unproductive immune responses in the context of whole-cell cancer vaccines [45]. Additionally, the research explored the utilization of fetal bovine growth supplements in cell cultures for investigations related to whole-cell vaccine research and manufacturing. The findings of the study were encouraging, indicating that whole-cell cancer vaccines have the capacity to induce long-lasting remissions and hold promise for advancement into late-stage clinical trials. Moreover, the study highlighted a potential strategy for enhancing vaccine efficacy by removing or reducing the presence of fetal bovine serum. These insights shed light on the potential of whole-cell cancer vaccines and provide valuable considerations for future research and development in the field [45].

The utilization of whole tumor cells as a vaccine offers a unique advantage compared to targeting a single protein or peptide tumor antigen. This approach allows for the presentation of all potential antigens expressed by cancer cells, removing the need to identify the optimal antigen for a specific cancer type. By exposing the immune system to the entirety of the tumor cell, whole-cell vaccines can elicit a broader immune response that involves the activation of T cells, B cells, and NK cells. Consequently, this approach holds great promise for the development of more efficient and adaptable cancer vaccines [31].

Despite the increasing incidence and mortality rates associated with melanoma, conventional treatments, such as chemotherapy and radiotherapy, have shown limited success in improving the overall survival of high-risk melanoma patients. However, the development of the allogeneic whole-cell melanoma vaccine, known as AGI-101H, has offered promising outcomes. Originally composed of autologous melanoma cells combined with two modified allogeneic cell lines expressing interleukin 6 (IL-6) and its soluble gp80 receptor (sIL-6R), early clinical studies in human melanoma patients were initiated in 1995, marking one of the pioneering gene therapy clinical trials globally. Notably, complete and partial clinical responses, as well as long-term survival, were observed. Subsequently, due to challenges in obtaining sufficient autologous melanoma cells, the vaccine composition was modified to include allogeneic melanoma cell lines. The current iteration of AGI-101H incorporates two melanoma cell lines retrovirally transduced with a designer cytokine gene known as Hyper-IL6 or H6, which serves as a molecular adjuvant. 

Moreover, chronic exposure of vaccine cells to H6 in an autocrine manner activates the Janus Kinase 1-Signal Transducer and Activator of Transcription 3-Octamer-Binding Transcription Factor 4 (JAK1-STAT3-OCT4) pathway, resulting in a shift towards a melanoma stem-cell-like phenotype or induced stem cell phenotype. Immunization of advanced melanoma patients with AGI-101H has demonstrated a significant increase in overall survival compared to traditional chemo- and radiotherapeutic approaches [46]. Similar promising results have been observed in renal cell carcinoma and prostate cancer models, where modified TME cells with the H6 adjuvant led to improved clinical outcomes and enhanced anti-tumor immune responses [47,48].

A study conducted by Nawrocki et al. (2001) revealed significant humoral responses, with approximately 50% of patients showing immunoglobulin G (IgG) responses to allogenic melanoma cells and 40% of patients responding to autologous cells. Intriguingly, since 1997 Nawrocki has achieved success in treating stage IV melanoma patients using genetically modified cellular vaccines (GMTV). Out of 16 patients, a clinical response was observed following GMTV immunization, and in 4 patients, complete regression of metastases was observed [49]. These findings demonstrate the potential of modified melanoma vaccines in eliciting immune responses and achieving positive clinical outcomes in melanoma patients. Phase II trials were conducted by Mackiewicz et al. (2012) to determine the efficacy and toxicity of adjuvant treatment using the Hyper-IL-6 gene-modified whole-cell allogeneic melanoma vaccine in patients with stage 3 and 4 resected diseases [50]. While there was minor toxicity observed related to local vaccination reaction, redness, edema, and itching at the injection site, there were no grade 3 or grade 4 toxicities observed. In fact, there was a significant increase in disease-free survival and overall survival of patients with continuous vaccination methods [50].

Przybyla et al. (2021) conducted a study highlighting the spontaneous development of CD8+ T cell responses to melanoma-associated antigens in healthy individuals [51]. These antigens, including Tyrosinase, MAGE-A3, Melan/Mart-1, gp100, and NY-ESO-1, are regularly expressed by normal melanocytes. The research findings suggest that healthy individuals possess natural autoimmunity directed against melanocytes, which may provide protection against the progression to malignant melanoma. This phenomenon was also observed in studies involving breast cancer patients, where healthy women exhibited increased levels of spontaneous T cell autoreactivity to HER-2, while women with breast cancer were found to lack this cellular response [52,53]. These findings shed light on the complex interplay between the immune system and cancer development, suggesting the potential role of pre-existing immune responses in preventing malignant transformations.

Even more recently, Kwiatkowska-Borowczyk et al., (2018) revealed that immunization of patients resulted in the generation of cytotoxic CD8+ T cells specific for Aldehyde Dehydrogenase 1 Family Member A1 (ALDH1A1) along with the production of specific anti-ALDH1 antibodies [54]. Kazimierczak et al., (2020) discovered a new biological marker for monitoring melanoma immunotherapy, where there is a positive correlation between the production of antibodies to BNIP3L/NIX and the clinical outcome of melanoma patients treated with the AGI-101H vaccine [55]. Increased expression of BNIP3L is also positively correlated with patient overall survival (OS) in melanoma. BNIP3L is induced by tumor suppressor p53 and, in response to hypoxic TME conditions, plays a crucial role in the clearance of damaged mitochondria with mitochondrial autophagy [55].

Further research conducted in 2015 by Mackiewicz et al. discussed the efficacy of AGI-101H as a candidate for the combination treatment of non-resected advanced melanoma. Immune checkpoint inhibitors or tumor hypoxia normalization agents are the perfect combination approaches to be added to AGI-101H vaccine therapy. Furthermore, patients that were treated in an adjuvant setting and received reinduction with AGI-101H showed a 70% reduction in risk of death compared with patients not reinduced [48]. Throughout the entirety of AGI-101H trials conducted in both adjuvant and non-resected melanoma contexts, involving a participant pool exceeding 400 patients who collectively received 40,000 vaccine doses, solely anticipated grade 1 and 2 toxicities were observed. In contrast to alternative treatment strategies like IL-2, INF-alpha, ipilimumab, nivolumab, or anti-PD-1L, which have been associated with severe grades 3 and 4 toxicities carrying the potential for life-threatening consequences, AGI-101H offers a markedly favorable safety profile [48,50,56].

Recent experimental studies in mice models demonstrated the increased effectiveness of the melanoma vaccine modified with H6 and admixed with murine melanoma stem-like cells (MSC) or induced mice pluripotent stem cells (miPSCs). The above vaccine significantly inhibited tumor growth and extended disease free-survival and OS compared to the vaccine alone in animal models. The MSC and miPSC additions increased the stemness of the basic vaccine, thus decreasing the local TME immunosuppression.

Despite noteworthy progress in cancer immunotherapy, our comprehension of what constitutes a favorable immune response beyond the AGI-101H melanoma cancer vaccine remains incomplete. It is imperative to investigate the factors contributing to a positive response and the underlying reasons for the substantial variations in the response among different patient populations. Moreover, continued research is necessary to explore the interaction between whole-cell vaccination and the TME. Such research has the potential to provide a more comprehensive understanding of immune responses and further enhance the effectiveness of cancer immunotherapy.

### 4.2. Induced Pluripotent Stem Cell-Based Vaccines

In 2006, Shinya Yamanaka made a groundbreaking discovery by developing iPSCs, building upon earlier work by Sir John Gurdon in 1962. Gurdon successfully reprogrammed the somatic cells of tadpoles into pluripotent embryonic cells, paving the way for subsequent cloning experiments [24].

iPSCs hold immense potential as a valuable tool in personalized medicine due to their ability to be derived from a patient’s own cells, ensuring compatibility and reducing the risk of immune rejection. In the field of cancer vaccine development, iPSCs play a crucial role. They can be generated by reprogramming adult somatic cells and exhibit gene expression profiles similar to embryonic stem cells. These iPSCs possess unique characteristics, combining the immunogenic properties of TME-extracted cells with the ability to proliferate in culture. Additionally, iPSCs possess the remarkable capability to differentiate into various germ cell lines, including endoderm, mesoderm, and ectoderm, offering a diverse range of cell types that can be utilized for innovative immunotherapeutic strategies [24].

iPSC vaccines offer a promising approach to precisely target the TME by engineering induced pluripotent stem cells. Through manipulation, iPSCs can be directed to generate TME-specific antigens found on various cell types within the TME, such as TAFs (transcription factors associated with the TATA-binding protein), endothelial cells, and immune cells. By presenting these TME-specific antigens, iPSC vaccines elicit an immune response that specifically targets the components of the TME. Moreover, iPSCs can be engineered to express immunostimulatory molecules or cytokines, enhancing the activation of immune cells and fostering an improved anti-tumor immune response within the TME. By expressing TSAs, iPSC vaccines activate T cells, including cytotoxic CD8+ T cells, which recognize and eliminate tumor cells within the TME. Consequently, iPSC vaccines provide a targeted and potent strategy for combating cancer within the complex TME.

Studies have demonstrated the effectiveness of iPSC vaccines in preventing tumor growth in genetically comparable cancer models. Notably, iPSC vaccines have shown indications of humoral and cellular immune responses when used prophylactically. They can reduce metastatic tumor burden, modify immune responses by influencing the balance of Th17 cells, and increase the infiltration of Gr1+CD11b+ myeloid cells into tumors. Remarkably, studies by Kooreman et al. (2018) have shown that unvaccinated recipients receiving T cells from vaccinated tumor-bearing mice exhibit an antigen-specific anti-tumor response [57].

More recently, Kishi et al. (2021) demonstrated the potential of iPSCs in combination with a histone deacetylase inhibitor (HDACi) called Valproic Acid (VPA) [58]. This combination improved the survival rate, reduced tumor volume, and transformed an immunosuppressive TME into an immune-activated TME in mouse models of aggressive triple-negative breast cancer cell lines. This research study’s main objective was to use iPSCs to target cancer stem cells otherwise resistant to conventional therapies. However, it is worth noting that iPSC production can be time-consuming, taking several months, and there is a risk of teratoma formation once injected, as iPSCs are immature progenitor cells [22,57].

### 4.3. In Situ Cancer Vaccines

In the early 2000s, Dr. Cornelis Melief and his colleagues at Leiden University Medical Center in the Netherlands pioneered a groundbreaking achievement: the development of the first in situ cancer vaccine. Named TriMix, this vaccine was specifically designed to activate and mobilize DCs within the TME. By doing so, it effectively stimulated an immune response against cancer cells. TriMix works by presenting TSAs to the immune system, triggering a targeted immune response against the cancer cells dwelling in the TME. This pioneering breakthrough laid the foundation for subsequent advancements in the field of in situ cancer vaccines. Since then, a wide range of in situ cancer vaccines have been developed and rigorously tested in both preclinical and clinical studies, further expanding our understanding of their potential in the fight against cancer.

Subsequent studies have revealed the remarkable potential of in situ cancer vaccines to address the immunosuppressive TME. Research conducted by Locy et al., in 2018 demonstrated that these vaccines possess the unique ability to convert various aspects of the TME, enabling effector T cells to infiltrate the tumor site and effectively eliminate cancer cells [19]. A key attribute of these vaccines lies in their capacity to induce distinct patterns of cytokine secretion. As discussed by Lurje et al., in 2021, this capability leads to diverse profiles of tumor-infiltrating lymphocytes (TIL), including variations in their type, quantity, and activation status [59]. Such heterogeneity culminates in immunogenic cell death (ICD), a process crucial for mounting a potent immune response against cancer. These significant findings highlight the transformative potential of in situ cancer vaccines in reshaping the immune landscape within tumors, thereby bolstering the body’s ability to combat cancer cells.

In situ cancer vaccines offer a distinct advantage by eliciting a potent and specific immune response against tumors while mitigating many of the systemic side effects commonly associated with traditional chemotherapy and radiation treatments. One remarkable attribute of these vaccines is their ability to generate the immune response directly within the TME, thereby holding the potential to effectively target and eliminate metastatic cancer in distant sites.

Recent research conducted by Wang et al., in 2020 sheds light on another significant benefit of in situ cancer vaccines. Their study reveals that an in-situ gel vaccine induces a process known as TAM repolarization, shifting these cells toward the M1 phenotype. This repolarization is associated with enhanced efficacy of the anti-tumor vaccine and prolonged survival in both preclinical and clinical settings [60].

### 4.4. Microbial Vector Vaccines

The concept of microbial vector cancer vaccines traces its origins back to the late 19th century and the mid-20th century. In 1891, the groundwork for bacterial-based vaccines was laid, followed by advancements in viral-based vaccines in the 1950s. These pioneering developments paved the way for the utilization of microbial vectors in cancer vaccine research. Microbial vector vaccines have emerged as a compelling approach due to their ability to stimulate antigen presentation through both MHC class I and class II pathways. This dual mechanism enhances the immunogenicity of these vaccines [61,62]. Live vector-based vaccines, a subset of microbial vector vaccines, consist of recombinant viral and bacterial vectors. These vectors are designed to carry the required antigens of the vaccine, replicate within host cells, and elicit robust immune responses against the targeted disease.

#### 4.4.1. Viral-Based Vaccines

In 1972, a significant milestone was achieved by Jackson et al., who successfully generated recombinant DNA from the SV40 virus. This breakthrough paved the way for further exploration and utilization of viral-based vaccines, particularly through the development of the vaccinia virus vector. Subsequently, a diverse array of novel vectors emerged and underwent evaluation in clinical trials, expanding the repertoire of viral-based cancer vaccines [63].

Viral-based vaccines employ genetically modified viruses as delivery vehicles, specifically targeting cancer cells while sparing healthy cells. Upon infiltrating cancer cells, these viruses replicate and express TAAs, which are then presented to immune cells. By presenting TAAs to the immune system, viral-based vaccines can elicit a potent immune response against cancer cells expressing these antigens. This approach harnesses the power of the immune system to selectively recognize and eliminate cancer cells, offering a highly targeted strategy for cancer immunotherapy. As emphasized by D’Alise et al. in 2022, viral-based vaccines hold significant potential to provide an effective and precise approach to combating cancer [64]. Among the most extensively studied vectors today are adenovirus and vaccinia virus. These vectors have demonstrated remarkable immune-stimulating properties, particularly in inducing the activation of CTLs, without necessitating the use of adjuvants [25].

Oncolytic virotherapy represents a compelling approach to enhance the effectiveness of cancer vaccines by orchestrating the modulation of the TME and selectively targeting and eliminating malignant tissue while preserving normal cells and surrounding tissues [65]. The oncolytic virus strategy in immunotherapy capitalizes on the ability of viruses to infect and replicate within tumor cells, resulting in cell death and the subsequent release of antigens and viral remnants. Crucially, the behavior and impact of the vaccine are determined by the vector and viral components’ capacity to stimulate the innate immune response, triggering interferon production and cytokine release [25]. The lysis of cancer cells leads to the liberation of various immunogenic components, including pathogen-associated molecular patterns (PAMPs), damage-associated molecular patterns (DAMPs), viral proteins, nucleic acids, TAAs, and immunogenic neoepitopes. These released factors initiate a cascade of immune responses, contributing to the activation of NK cells, which target cancer cells exhibiting reduced expression of MHC I. This process also induces ICD, further bolstering the immune response. Moreover, these cascades foster the initiation of de novo T cell responses against TAAs and neoantigens [22]. This multifaceted immune activation provides a robust defense mechanism against cancer, leveraging the innate and adaptive immune systems to combat malignant cells.

There has been a recent proposal for a new concept of oncolytic viral therapy where the virus is engineered in hybrid vectors to circumvent the different side effects of individual viral strains. In a particular study by Martínez-Vélez et al., (2019), the potential of the oncolytic adenovirus Delta-24-RGD elicits an anti-tumor effect on a variety of pediatric glioma [66]. Most preclinical studies within the past 10 years have focused on the HSV1716 oncolytic herpes virus treatment for neuroblastoma. In all studies, the oncolytic virus displayed no toxicity. While there are minimal studies on oncolytic adenovirus for the treatment of high-grade pediatric brain tumors, Delta-24-RGD has started clinical trials, demonstrating safe measures and efficacy against gliomas [66].

Some limitations of viral-based cancer vaccines can be dependent on pre-existing immunity to the virus used in the vaccine, which can reduce the effectiveness of the treatment. There is also a risk of adverse effects from the viral vector used in the vaccine, such as inflammation or allergic reactions. Despite these limitations, viral-based cancer vaccines have shown some effectiveness in preclinical and clinical studies; for example, the FDA has approved the use of a viral-based cancer vaccine called Sipuleucel-T for the treatment of advanced prostate cancer. Clinical trials are ongoing for other types of cancer, including lung cancer, melanoma, and glioblastoma. To improve the effectiveness of viral-based cancer vaccines, researchers are exploring various strategies, such as combining the vaccine with other immunotherapies targeting multiple antigens and optimizing the dosing and timing of the treatment.

Viral vaccines have emerged as powerful tools in targeting tumor angiogenesis, leading to regression or impeding the progression of distant metastases from the site of administration. Notably, talimogene laherparepvec, an FDA-approved oncolytic virus based on herpes simplex virus type 1 (HSV-1), marketed as T-VEC (ImlygicTM), has shown efficacy in treating surgically unresectable metastatic melanoma. Another promising viral vaccine in this category is PSA-TRICOM (Prostvac-VF), currently undergoing clinical trials (NCT02326805) for advanced prostate cancer [22,26]. The PSA-TRICOM platform employs a strategic approach wherein it enables the expression of prostate-specific antigen, a marker for prostate cancer while incorporating three T cell receptor-stimulating co-regulators (TRICOM). This incorporation facilitates the activation of previously dormant DCs and T cells within the TME, as explained by Thomas and Prendergast in 2016 [26].

It is important to note that, due to in situ virus replication, the viral dose needs to be incrementally increased over time, and adherence to a scheduled vaccine protocol is imperative. These considerations ensure optimal virus-mediated therapeutic outcomes and sustained immune activation.

#### 4.4.2. Bacteria-Based Vaccines

Long before the discovery of viral-based vaccines was the use of bacteria-based vaccines for cancer immunotherapy. As mentioned previously, in 1891, William Coley successfully used mixtures of live and inactivated *Streptococcus pyogenes* and *Serratia marcescens* in a novel treatment of sarcoma, leading to tumor regression [62]. Coley proved that the diverse use of bacteria-based vaccine vectors can be used to vaccinate against variable TAAs, deliver cytokines, and target immunosuppressive molecules. Elimination or conversion using chemotherapeutics or attenuated *Listeria*, along with tumor-killing agents to the TME using bacterial vectors, have been known to increase the effectiveness of cancer vaccines in older age patients [67].

In specific subsets of patients, the utilization of bacteria-based vaccines has demonstrated the capacity to generate long-term immunity and effectively target metastases. This remarkable ability, coupled with the potential to impede tumor proliferation, prevent metastasis, and hinder disease recurrence, positions bacteria-based vaccines as a formidable alternative to conventional therapies [60]. One promising strategy lies in directing the bacteria to the TME, a mechanism that holds significant potential in reducing systemic toxicity and enhancing therapeutic efficacy. By precisely delivering the vaccines to the TME, bacteria-based vaccines can more effectively engage with the tumor cells and trigger an immune response with heightened specificity.

It is important to acknowledge that the mechanisms and pathways employed by these vaccines can vary significantly, necessitating a deeper understanding of the future development of next-generation vaccines. As we delve further into their intricate workings, researchers can unlock new insights and leverage this knowledge to refine and advance the field of cancer vaccines.

Bacterial vectors, such as *Listeria monocytogenes* (Lm), *Lactobacillus casei* (*L. casei*), *Lactobacillus lactis*, and *Salmonella*, have undergone clinical trials and demonstrated their ability to elicit both innate and adaptive immune responses. Importantly, these bacterial vectors have shown the potential to reduce systemic toxicity, thereby enhancing therapeutic efficacy. By modifying the bacterial vectors to express cytokines and tumor antigens (TAs), such as IL-2 or mesothelin, they can induce targeted immune responses from T and NK cells that specifically recognize tumor cells [22]. One notable advantage of using bacterial vectors is the potential to simplify and economize the manufacturing and vaccination process by circumventing the challenges associated with target antigen purification. This approach offers a straightforward and cost-effective means for the large-scale production and administration of vaccines [60].

It is worth noting that certain bacteria can pose a risk factor for specific types of cancer. Among the well-known examples is *Helicobacter pylori*, which is strongly associated with gastric cancer [68]. Additionally, bacterial infections caused by *Chlamydia trachomatis*, *Neisseria gonorrhoeae*, and *Treponema pallidum* (syphilis) have been linked to an increased risk of developing cervical cancer.

### 4.5. Peptide Vaccines

While the focus of immunization has been largely based on infectious diseases and the treatment of allergies, current immunization efforts focus on noninfectious diseases, primarily directed against cancers. In fact, most cells in the TME are differentiated based on the regulation of endogenous proteins or mutations in those proteins, being a suitable target for vaccines [27].

The main goal of peptide immunogen is to induce protective T cell and B cell immunity. The earliest peptide vaccination study came from virus-derived CD8+ T cell epitopes, which was reported in the late 1980s, and discovered that mice vaccinated with small synthetic peptides can be recognized by CD8+ CTL [27]. Polypeptides and protein-based vaccines can significantly activate T cells resulting in a heightened immune response and enhanced T cell activation. They also sporadically fail to induce memory CD8+ T cell responses [22,26]. Peptide-based vaccines can be composed of TSAs, TAAs, CGAs, and virus-associated antigens; however, the most distinct categories tackled in this review include TAA and TSA [22].

Synthetic peptides used for the production of these vaccines usually consist of 20–30 amino acids that target tumor antigen-associated epitopes. In comparison to genetic vaccines, peptide vaccines are not able to encode full-length tumor antigens [69]. Most peptide vaccines can be delivered in the form of synthetic T cell epitopes or in combination with T cell and B cell epitopes into one therapeutic preparation. Synthetic T cell epitopes bind to HLA classes I and II of the APCs stimulating CD8+ T cells or CD4+ T helper cells to target TAAs or TSAs. These HLA-peptide complexes activate immune responses from CTLs and T helper cells. Another approach is using whole proteins as antigen carriers, namely liposomes, microemulsions, immune-stimulating complexes, and other microparticle systems.

Peptide cancer vaccines work by targeting specific TAAs expressed by cancer cells within the TME. These vaccines consist of short peptide sequences that mimic the TAAs and are administered to stimulate an immune response against the cancer cells. When peptide cancer vaccines are introduced into the TME, they are processed by APCs, such as DCs. The APCs internalize the peptides and present them on their surface in complex with MHC molecules. This presentation allows the peptides to be recognized by T cells, specifically CD8+ CTLs, which are capable of directly killing cancer cells expressing the targeted TAAs. The interaction between the presented peptide-MHC complex and the T cell receptor (TCR) triggers the activation of the CTLs. This activation leads to the expansion of a population of tumor-specific CTLs that can specifically recognize and target cancer cells expressing the TAAs. These CTLs can infiltrate the tumor and exert their cytotoxic effects, thereby combating the cancer cells within the TME [70].

Recent research has highlighted the potential of an IDO-specific (indoleamine 2,3-dioxygenase-specific) peptide vaccine as a targeted therapeutic strategy within the TME. This innovative vaccine specifically aims to address the immunosuppressive effects of IDO+ cells, a key component in myeloid-originated tumors. By inducing an IDO-specific immune response, the vaccine effectively targets and reduces the number of these suppressive cells while simultaneously increasing the presence of CD8+ T cells. These encouraging results suggest that combining this vaccine with other cancer vaccines may amplify their efficacy [71].

Peptide vaccines offer several advantages, including ease of synthesis, low production costs, low carcinogenic potential, and high chemical stability. However, they may exhibit low immunogenicity, and thus, combination strategies should be considered [18,69]. To enhance their effectiveness, computer-based algorithms can be employed to screen amino acid sequences and identify those with the greatest compatibility. Experimental testing of the selected sequences for antigen specificity is also essential [71]. It should be noted that while peptide vaccines have shown the ability to delay immune responses and inhibit tumor growth, significant tumor shrinkage has yet to be consistently demonstrated [69].

When discussing peptide vaccination, delivery of the peptide antigen becomes a crucial consideration due to its potential toxicity. Li et al. (2014) have extensively explored various administration routes, including transdermal patches, subcutaneous injection, and intravenous injection, assessing their safety and efficacy [27].

The transdermal route of administration has been the safest and commendable by being needle-free and eliminating the need for healthcare professionals to administer the vaccine. Particularly, this can help with systemic immunity and increase shelf life and stability while reducing the cost of application [72]. Regarding the subcutaneous injection and intravenous injection of a peptide vaccine, it was determined that these methods were also safe by detailing the immediate adverse effects within 30 min of administration.

Regardless of the several advantages, peptides are typically more immunogenic when used with next-generation adjuvants than without. Future development and improvement of the safety concerns related to the use of adjuvants and particulate peptide vaccine delivery systems should be considered. The specific limitations of older adjuvants include a lack of cellular immune stimulation, degradation on freeze drying, and the possibility of adverse local reactions; thus, newer developments in the delivery system focus on overcoming these imperfections [73,74]. Substances that create adverse reactions, like toxins, lipids, and lipopolysaccharides, are typically avoided in the development of this vaccine.

In recent trials, incorporating proteins or peptides in cancer immunotherapy has shown promising results, particularly in targeting novel cellular antigens, such as preferentially expressed antigens in melanoma (PRAME). PRAME is a cancer-testis antigen expressed in solid tumors and hematologic malignancies, with high expression correlating with a good prognosis in acute myeloid leukemia [75].

Pujol et al. (2016) conducted a phase I dose escalation study targeting PRAME antigens in clinical trials (NCT01159964). The study aimed to assess the safety and immunogenicity of an immunotherapeutic consisting of recombinant PRAME and the immunostimulant AS15 [76]. Sixty patients with PRAME-positive resected non-small cell lung carcinoma (NSCLC) were divided into three groups, receiving different doses of PRAME antigen immunotherapy. The study found detectable anti-PRAME antibodies in all patients after four doses, with the highest percentage of patients showing PRAME-specific CD4+ T cell responses in the group receiving the highest dose (500 μg). However, no predefined CD8+ T cell responses were detected, and further development of the immunotherapeutic was halted when comparable results were not achieved in NSCLC patients [76].

A specific subtype of diffuse glioma is characterized by a mutation in isocitrate dehydrogenase 1 (IDH1), with the most common mutation affecting codon 132, resulting in the IDH1(R132H) protein. In a study by Schumacher et al. (2014), an IDH1(R132H)-specific vaccine demonstrated efficacy in inducing a targeted T helper cell response against IDH1(R132H) + tumors in MHC-humanized mouse models [77].

To further investigate the safety and immunogenicity of the IDH1-specific vaccine in humans, a multicenter phase I trial was conducted on 32 patients with newly diagnosed grade 3 and 4 IDH1(R132H) + astrocytoma. The trial administered an IDH1 targeted peptide-based vaccine (IDH1-vac) containing 300 ± 30 μg of immunogenic peptide. The patients, both male and female, ranged in age from 18 to 65 years. The trial group was then divided into three subgroups based on their previous treatment: TG1 received radiotherapy alone, TG2 received three cycles of TMZ (temozolomide) chemotherapy alone, and TG3 received both radiotherapy and chemotherapy. Among the participants, 17 underwent complete tumor resection, 12 had a subtotal resection, and 3 underwent a biopsy. Methylation analysis categorized 14 patients as low grade and 10 as high grade. The IDH1-vac was administered periodically at specific weeks, and blood samples were collected for immunogenicity testing. MRI (magnetic resonance imaging) scans were performed at designated time points to monitor the patients’ response to the vaccine.

In a clinical trial (NCT02454634) evaluating the IDH1-specific vaccine in patients with IDH1(R132H) + astrocytoma, vaccine-related side effects were limited to grade 1, and no significant toxicity that would impact the treatment regimen was observed. Encouragingly, the vaccine-elicited immune responses in 93.3% of patients spanning multiple MHC alleles. T cell responses were detected in 26 out of 30 patients, while B cell responses were observed in 28 out of 30 patients. Analysis of the IDH1-vac-induced T cells using flow cytometry revealed the production of tumor necrosis factor (TNF), interferon-γ (INF-γ), and interleukin-17 (IL-17) by T helper cells.

Follow-up analysis showed favorable three-year progression-free and death-free rates, with 63% (95% CI 44–77) and 84% (95% CI 67–93) of patients experiencing no disease progression or death, respectively. Notably, patients who did not develop an immune response had a higher risk of disease progression within two years compared to those with an immune response, whose two-year progression-free rate was 82% (95% CI 62.3–92.1). These findings from Platten et al. (2021) underscore the safety, immunogenicity, and potential clinical benefit of the IDH1-specific vaccine in patients with IDH1(R132H) + astrocytoma. The vaccine-induced immune responses, particularly the T cell responses producing TNF, INF-γ, and IL-17, appear to be associated with improved clinical outcomes [6].

### 4.6. Nucleic Acid-Based Vaccines

Gene-based vaccines utilize DNA or RNA to deliver the coding region of an antigen, stimulating a host immune response and production of selected antigens [78]. These vaccines have several advantages. (1) They can encode full-length tumor antigens, allowing for the presentation of multiple epitopes and a broader T cell response. (2) Vaccination can activate DCs and increase pro-inflammatory cytokine levels. (3) Fusion genes can be produced to enhance the generation of T-helper memory response.

Choosing the right plasmid, typically of bacterial origin, is crucial in gene-based vaccine production. However, the main challenge lies in the delivery method [18,78]. While electroporation and viral vectors show high efficiency, they are difficult to use in clinical practice and may lead to variable immune responses and unwanted side effects.

Nucleic-acid-based vaccines offer the advantage of delivering multiple antigens and targeting various TAAs or somatic tumor mutations. This activation of both humoral and cell-mediated immune responses increases the chances of overcoming vaccine resistance. The two main categories of gene-based vaccines are DNA- and RNA-based vaccines.

#### 4.6.1. DNA Vaccines

DNA vaccines are plasmid-based vaccines that deliver genes encoding tumor antigens, triggering an innate immune response. The presence of unmethylated CpG motifs in the delivered DNA stimulates immune response pathways. DNA vaccines are known to elicit a potent CD8+ T cell response against neoantigens compared to RNA and peptide vaccines [22,28]. They are stable, cost-effective, and easy to produce and can be stored without strict cold chain requirements. 

To enhance targeted delivery, engineered cell-derived exosomes and synthetic nanoparticle complexes have shown promise [23,79]. Delivery techniques, such as electroporation, sonoporation, gene guns, or DNA tattooing, are commonly used. However, as a monotherapy, DNA vaccines have limitations and are insufficient to escape immune system recognition and attack [22].

To maximize efficacy, DNA vaccines are often combined with other strategies and adjuvants, such as cytokines, immune checkpoint blockade, chemotherapy, radiotherapy, and endocrine therapy [80]. However, concerns exist regarding the possibility of plasmid DNA integrating into the chromosome, the use of genes encoding cytokines or co-stimulatory molecules, and the potential undesirable effects of the expressed antigen itself [81].

#### 4.6.2. RNA Vaccines

RNA vaccines for cancer are a relatively new field, but the use of RNA as a therapeutic agent has been explored for some time. Early attempts to develop RNA vaccines encountered challenges with stability, immunogenicity, and delivery. However, considerable progress was made in the early 2000s, with researchers demonstrating the potential of RNA vaccines for cancer immunotherapy in animal models. They showed that injecting RNA encoding TSAs could induce a robust immune response and delay tumor growth [82]. Following this breakthrough, various companies and academic institutions began developing RNA vaccine candidates for different diseases. The culmination of these efforts came in 2020 when the first RNA-based vaccines for COVID-19 were authorized for emergency use. Developed by Pfizer-BioNTech and Moderna, these vaccines marked a major milestone in the history of RNA vaccines.

RNA-based cancer vaccines have gained significant interest in recent years, with numerous studies investigating their potential in treating diverse types of cancers. In a notable development, the U.S. FDA approved the first RNA vaccine for cancer, called “Imlygic” or talimogene laherparepvec, in 2020. This vaccine utilizes a modified herpes simplex virus to selectively replicate within cancer cells, causing them to rupture. It also releases GM-CSF, a protein that stimulates the immune system to target and attack cancer cells.

There are three main types of RNAs being studied as cancer vaccines: non-replicating unmodified mRNA, modified mRNA, and self-amplifying mRNA derived from viruses [83]. Naked RNA is unstable, so researchers have explored different delivery methods, such as the “gene gun”, protamine condensation, and encapsulation, to enhance stability and performance [22]. Unlike DNA vaccines, mRNA vaccines can be translated into both dividing and non-dividing cells, and they do not integrate into the genome sequence [82]. mRNA vaccines offer advantages such as high potency, safe administration, rapid development potential, and cost-effective manufacturing.

Cafri et al. (2020) conducted studies focused on the detection and selection of neoantigens expressed by autologous cancer and recognized by TILs [29]. Metastatic gastrointestinal tumors were harvested, and TILs were collected for analysis. Using high-throughput immunologic screening, tumor-specific mutations were sequenced, and TSAs were identified using long peptides and tandem minigenes. In this study, four patients with gastrointestinal cancer, who had previously been treated with TILs or anti-PD1 agents, were intramuscularly injected with a personalized mRNA-4650 vaccine. The vaccine contained up to 20 selected antigens, including mutations in TP53 (tumor protein 1 gene), KRAS (Kirsten rat sarcoma virus), or PIK3CA, as well as up to 15 in silico-predicted HLA class 1 potential neoantigens. Two patients received eight injections of 0.13 mg, while the other two received four injections of 0.39 mg. The patients experienced mild toxicities, mostly grade 1 and 2, which resolved quickly without severe adverse effects. The study observed that approximately 15.7% of the predicted antigens induced a T cell-specific immune response, with 59% of the epitopes being CD4+ and 41% CD8+ epitopes. The number of vaccination-induced mutations varied, ranging from 2 to 6 per patient. It is worth noting that one patient did not show evidence of an immune response following vaccination [29].

### 4.7. Exosome-Based Vaccines

Recent studies show that tumor-derived exosomes are highly enriched in tumor antigens, MHC molecules, heat-shock proteins, and inducible co-stimulatory molecules found in the TME. Exosomes can trigger the differentiation of fibroblasts into CAF, leading to elevated smooth muscle actin expression and angiogenesis with the promotion of VEGF, fibroblast growth factor (FGF), platelet-derived growth factor (PDGF), basic FGF, transforming growth factor β (TGF-β), TNF-α, and interleukin-8 (IL-8) [30]. This is a promising research area for vaccine development as they behave as signaling molecules in cancer promotion and TME remodeling [84]. While exosomes are also capable of delivering functional RNAs to target cells, in combination with immunostimulatory agents, they can trigger potent CD8+ T cell anti-tumor responses while serving as useful biomarkers in cancer screening and early diagnosis [23].

A hepatocellular carcinoma (HCC) study described in the Lou et al. (2015) article on exosome delivery of anticancer drugs across the blood-brain barrier demonstrated the delivery of chemotherapeutic agents, like 5-FU and sorafenib, via adipose tissue-derived mesenchymal stem cell (AMSC) exosomes. miR-122, a key regulator of liver physiology and disease biology, was modified for additional use in this exosome treatment to increase the chemosensitivity of HCC cells [85]. In zebrafish models described in the same article, brain endothelial-cell-derived exosomes could pass through the blood-brain barrier and deliver medications, like paclitaxel and doxorubicin, for the treatment of brain cancer [86].

Perhaps the greatest concern of exosome vaccination regarding the TME is tumor resistance to therapy. Epithelial-mesenchymal transition (EMT) is promoted by carrying factors, like TGF-β, HIF1α (hypoxia-inducible factor 1-alpha), β-catenin, IL-6, β-catenin or vimentin, casein kinase, and several miRNAs. Induction of EMT may lead to tumor resistance to therapy through anti-apoptotic pathway promotion via drug efflux or drug sequestration, signal transduction alteration, and immune cell modulation [87].

## 5. Delivery Methods

The efficacy of these vaccines relies not only on the selection of appropriate antigens but also on the development of efficient delivery methods. Several strategies have been employed to deliver cancer vaccines tailored to optimize immune responses and enhance therapeutic outcomes. Here, we discuss common delivery methods for cancer vaccines and provide examples of their application in clinical settings.

Injection-based delivery is the most widely utilized approach for cancer vaccines, allowing for direct delivery of the vaccine into the body. Intramuscular or subcutaneous injections are commonly used, such as in the administration of HPV vaccines like Gardasil and Cervarix, which help prevent cervical cancer and other HPV-associated malignancies [88,89]. Intratumoral injections have also been employed, particularly for localized tumors, to induce a potent immune response within the TME [90].

Intravenous (IV) infusion provides a systemic delivery route, enabling the distribution of cancer vaccines throughout the body. PROVENGE, an FDA-approved vaccine for advanced prostate cancer, is administered via intravenous infusion [91]. This vaccine utilizes autologous DCs loaded with a fusion protein to activate the patient’s immune system against prostate cancer cells.

Oral administration offers a convenient and non-invasive delivery method for certain cancer vaccines. An example is the oral polio vaccine, which has been repurposed for the treatment of glioblastoma. Known as PVSRIPO, this vaccine utilizes a modified poliovirus to target and kill tumor cells [92].

Topical application involves directly applying the cancer vaccine to the skin or mucous membranes. Talimogene laherparepvec (T-VEC), an oncolytic virus-based vaccine, is administered through direct injection into cutaneous melanoma lesions [93]. By selectively replicating within tumor cells, T-VEC leads to their destruction and the induction of a systemic immune response against the cancer.

Gene delivery represents another approach in cancer vaccine development, involving the delivery of genetic material encoding TSAs. For example, the ADVAXIS-HPV vaccine utilizes a live attenuated bacterium to deliver a tumor antigen (HPV E7) gene into the body. The genetic material is expressed by cells, promoting immune recognition and tumor-specific immune responses [94].

Electroporation enhances vaccine delivery by applying electric pulses to the skin or tumor site. This method increases cell membrane permeability, enabling efficient uptake of the vaccine. Clinical trials have utilized the TriGrid™ electroporation system to enhance the delivery of melanoma-specific vaccines, such as the gp100 peptide vaccine, resulting in improved immune responses [95].

Nanoparticle-based delivery offers a versatile platform for cancer vaccine administration, providing enhanced stability, targeted delivery, and controlled release of vaccine components. Various nanoparticle-based vaccines, including lipid-based nanoparticles, polymer nanoparticles, and virus-like particles, are being investigated. These systems can encapsulate tumor antigens, adjuvants, or genetic material, facilitating efficient antigen presentation and immune stimulation [96].

The development of effective cancer vaccines necessitates the utilization of appropriate delivery methods. The diverse range of delivery approaches, including injection, intravenous infusion, oral administration, topical application, gene delivery, electroporation, and nanoparticle-based delivery, allows for tailored immunization strategies. Ongoing research aims to optimize vaccine delivery, combining multiple strategies to elicit robust and durable anti-tumor immune responses, thereby improving therapeutic outcomes for cancer patients.

## 6. Improving Vaccine Outcomes

Since the first approved adjuvant, called alum, was developed in 1926 by Alexander Glenny, hundreds of materials have been studied as adjuvants. It consists of aluminum salts, such as aluminum hydroxide or aluminum phosphate. Alum adjuvants enhance the immune response by promoting the activation of APCs and the release of pro-inflammatory signals, leading to the recruitment and activation of immune cells. It also enhances the uptake and presentation of antigens to immune cells, thereby stimulating both antibody and cellular immune responses. In 1936, Freund’s complete adjuvant was developed but was found to induce severe local necrotic ulcers and is considered too toxic for human use; thus, alum quickly became the gold standard for efficacy. Leading from alum to cytokines, TLR agonists, saponins, mineral salts, emulsions, and bacterial components, like lipopolysaccharides and lipophile phospholipids, led to the development of less toxic and a more stable FDA-approved vaccination adjuvant [13] MF59, virosome, AS03, AF03, and monophosphorylate lipid A (MPLA) in conjunction with alum are just some of the newly approved adjuvants used.

TLRs are a type of pattern recognition receptor that recognizes specific molecular patterns on pathogens. TLR agonists mimic these patterns and activate the innate immune system, leading to increased antigen presentation, enhanced production of pro-inflammatory cytokines, and improved activation of adaptive immune responses. Various TLR agonists, such as MPLA, can serve as adjuvants to improve vaccine efficacy. An example of the use of a liposome as an adjuvant is the peptide-CpG-liposome composite vaccine developed by Park et al. (2018). It was observed to induce humoral responses and inhibit cancer growth in pancreatic cancer, metastatic hepatocellular carcinoma, and colon cancer murine tumor models [97].

MPLA is a chemically modified derivative of lipopolysaccharides that displays reduced toxicity while maintaining the immunostimulatory activity seen in lipopolysaccharides [5,98]. Because they are lipid-based vesicles, they can encapsulate antigens and deliver them to immune cells. They can function as adjuvants by improving antigen presentation, enhancing antibody production, and promoting cellular immune responses. Liposomes can be designed to carry various immune-stimulating molecules, such as CpG DNA, which activates TLR9 and triggers immune activation.

We see MPLA used specifically in the HBV (hepatitis B virus) vaccine Fendrix for patients with renal insufficiency and in broad HPV vaccines like Cervarix. With a history of safe and effective use, MPLA technology has shown wide success in clinical trials and is being considered for peptide vaccine delivery. It is important to note that the effectiveness of MPLA as an adjuvant in targeting the TME can depend on numerous factors, including the specific cancer type, the stage of the disease, and the overall immune status of the individual. Combination approaches using MPLA with other immunotherapeutic agents or treatments may also be explored to further enhance the anti-tumor immune response and target the TME more effectively.

Saponins, extracted from the Quillaja saponaria plant (QS-21), function as immunostimulant adjuvants having strong anti-inflammatory properties. QS-21 is one of the most widely used adjuvants in vaccines. Saponins can induce the activation of APCsing cells, stimulate the production of pro-inflammatory cytokines, and promote the generation of both humoral and cellular immune responses.

GM-CSF is commonly used as an immunostimulatory adjuvant, where cytokines direct the differentiation, proliferation, and activation of macrophages and DCs with a focus on cDC1 and Th1 responses [99]. GM-CSF induces an influx of immune cells, including DCs, macrophages, eosinophils, and T cells, at the vaccination site, which is crucial for initiating and orchestrating immune responses against pathogens or tumors.

GM-CSF cytokines have been found to be more effective than other researched cytokines, such as IL-2, IL-4, IL-5, and y-IFN, as it activates a tumor-specific T cell response and is used in the production of GM-CSF-secreting whole-cell cancer vaccines [31,100,101]. The GM-CSF vaccine adjuvant produced conflicting results in clinical trials, some showing only a weak effect in enhancing the immune response of the vaccine [13]. In others, there were no positive effects at all [102,103]. However, high clinical efficacy has been found in combination with other immunotherapy [103].

These different adjuvants work through various mechanisms to enhance the immune response, including promoting antigen uptake and presentation, activating innate immune cells, inducing the production of pro-inflammatory cytokines, and directing immune responses towards a desired profile (e.g., Th1 responses). By improving the immune response, adjuvants help to generate stronger and more long-lasting protective immunity against pathogens or antigens of interest.

It is important to note that the effectiveness of adjuvants can vary depending on the specific vaccine formulation, target pathogen or antigen, and the characteristics of the immune system in individuals. Combination approaches using multiple adjuvants or adjuvants in conjunction with other immunotherapeutic strategies are also being explored to further enhance the immune response and target specific immunological pathways effectively.

### Radiotherapy, Chemotherapy, and Naturopathy

In preclinical studies conducted by Adler et al. (1998), vaccines given five weeks after radiotherapy were most effective [104]. Recent studies have observed an increase in cancer cell resistance to chemotherapy and radiotherapy and the promotion of tumor angiogenesis, leading to a greater risk for tumor invasion and metastasis [105].

Some clinical responses can be rapid, being active and efficient within just a few weeks with fast, noticeable regression. However, in comparison to chemotherapy and radiotherapy, where the response can take 2–3 months, therapeutic vaccine methods alone may be considered as having a slower immune response and could take several months to be effective. With several studies and assessments, it was determined that treatment methods like radiotherapy and chemotherapy added to the adjuvant show a higher success rate in treatment. In pancreatic ductal adenoma (PDA), discussed in a study by Mandili et al. (2020), chemotherapy enhances the immune response to TAA [106]. Vaccinations combined with Ipilimumab, for example, could increase the density of proliferating intratumoral CD8+ T cells or ISCOMATRIX (a saponin-based adjuvant). Ideally, this would develop a strong CD4+ and CD8+ T cell response [107,108].

With clinical trials, Cuzzubbo et al. (2021) also described the importance of using a more naturopathic method to help increase immune system strength, which could lead to increased effectiveness in cancer vaccines [13]. They explained that since radical treatments like chemotherapy and radiotherapy could strongly weaken the immune system, changing diet, increasing physical effort, and reducing stress could increase the effectiveness of anticancer vaccines [13]. Webber et al. (2018) stated that a ketogenic diet may produce clinical application and described its effectiveness as being similar to adjuvant therapy in cancer patients. They also mentioned how the effectiveness and safety of the ketogenic diet as supplementary therapy depended on the tumor location and genotype [109]. This could be something to consider during treatment.

## 7. Limitations of Cancer Vaccines

Despite the immense potential for cancer vaccination, various limitations hinder the widespread application and optimal efficacy of these vaccine types. The following presents an overview of the limitations associated with each vaccine category, shedding light on the challenges they pose in clinical translation.

Cell-based cancer vaccines encounter several limitations that hinder their widespread adoption and effectiveness. The process of isolating and culturing patient-specific immune cells demands significant resources and expertise, leading to high production costs that strain healthcare systems and limit patient access to this personalized therapy. This necessity for individualized vaccines tailored to specific tumor antigens poses challenges in mass production and distribution, impacting the scalability and logistical feasibility of these vaccines. Moreover, tumors can establish suppressive microenvironments that impede the activated immune cells’ efficacy, hindering their ability to recognize and combat tumor cells effectively. Tumor cells may thus develop immune evasion mechanisms, further compromising the overall efficacy of the vaccine and facilitating tumor progression. Additionally, the transportation and storage of cell-based vaccines under stringent conditions pose logistical obstacles, potentially jeopardizing the viability of these vaccines during distribution [110].

Ethical considerations constitute one of the primary concerns regarding iPSC-based vaccines. The process of generating iPSCs may involve the use of embryonic stem cells and genetic manipulation, raising ethical questions that demand careful evaluation before proceeding with clinical applications. The safety profile of iPSCs is a critical aspect that requires extensive preclinical safety assessments to address the potential risks of tumor formation or unwanted cellular responses. Additionally, like other vaccine types, iPSC-based vaccines may encounter immune evasion challenges, impacting their ability to provoke robust anti-tumor immune responses [111].

The identification of specific tumor antigens for in situ cancer vaccines can be a formidable task, particularly for cancers characterized by high genetic heterogeneity. The intricate TME may pose immune suppression, diminishing the immune response elicited by in situ vaccines and consequently reducing their overall efficacy. Furthermore, they might be limited to localized tumor sites, potentially offering suboptimal effectiveness against metastatic tumors [112,113].

Pre-existing immunity to the viral vectors used in microbial vector vaccines can compromise their effectiveness, particularly in individuals who have been previously exposed to the vector. Safety concerns associated with certain microbial vectors need to be thoroughly addressed to prevent adverse immune responses in vaccinated patients. Additionally, the limited cargo capacity of microbial vectors poses a challenge in delivering multiple tumor antigens simultaneously, potentially limiting the scope of immune responses generated by the vaccines [114].

The delivery of nucleic acids (DNA or RNA) to target cells demands specialized delivery systems or technologies to ensure efficient and effective transfection. The transient expression of antigens encoded by nucleic-acid-based vaccines may require multiple doses to sustain an adequate and prolonged immune response. Furthermore, the development of immune tolerance to the encoded antigens can limit the vaccine’s long-term efficacy, necessitating strategies to mitigate immune tolerance and improve vaccine durability [115].

Peptide-based cancer vaccines encounter several other limitations that impact their efficacy and applicability. Their focus on specific tumor antigens may lead to the oversight of other relevant antigens that could be targeted by the immune system, thereby limiting their overall effectiveness in eliciting a comprehensive anti-tumor response. Additionally, the requirement for peptides to bind to specific HLA molecules for immune recognition restricts their use in patients with compatible HLA types, potentially excluding a significant proportion of the patient population. The genetic diversity of tumors further complicates the efficacy of peptide vaccines, as some tumor cells may lack the targeted peptides, leading to suboptimal immune responses. Compared to whole-cell-based vaccines, peptide vaccines may induce weaker immune responses, necessitating the implementation of supplementary strategies to enhance their immunogenicity. Lastly, the incorporation of adjuvants or immune-stimulating agents is often necessary to potentiate the immune response elicited by peptide vaccines, resulting in more complex vaccine formulations [116].

The scalability of exosome production for exosome-based vaccines is a challenge that requires careful optimization to ensure sufficient vaccine availability for large-scale therapeutic use. Efficient loading of exosomes with an adequate amount of tumor antigens is essential to maximize their immunogenicity and enhance their potency as vaccines. Moreover, exosomes’ potential to modulate the immune response may have unintended consequences on vaccine efficacy and safety, necessitating a comprehensive understanding of their interactions with the immune system.

## 8. Conclusions

Cancer vaccines represent a promising avenue in the fight against cancer, harnessing the power of the immune system to prevent tumor growth, recurrence, or metastasis while enhancing its ability to recognize and eliminate cancer cells. The intricate composition of TMEs and the diverse responses they elicit play a crucial role in determining treatment outcomes. The activation of T cells is vital for effective immune responses against cancer, while B cells contribute to both tumor suppression and promotion. NK cells hold the potential to eliminate tumor cells but face challenges within the immunosuppressive TME. DCs play a crucial role in antigen presentation and T cell activation but may be impaired by tumor-derived factors. Neutrophils and TAMs exhibit dynamic roles, capable of switching between pro-tumor and anti-tumor states, influencing tumor initiation and progression. Understanding the complex interactions within TMEs is essential for designing effective cancer vaccines.

Delivery methods and the inclusion of adjuvants have proven pivotal in optimizing cancer vaccine efficacy. Various delivery approaches, such as peptide-based, nucleic acid-based, protein-based, viral vector-based, and DC-based vaccines, offer distinct advantages and challenges that need to be considered for successful implementation. The integration of adjuvants, such as TLR agonists, cytokines, and immune checkpoint inhibitors, enhances immune responses and promotes sustained immune activation, further augmenting vaccine efficacy.

Overcoming the challenges associated with cancer vaccines requires a multifaceted approach driven by innovative research and clinical advancements. To enhance the accessibility and affordability of cell-based vaccines, streamlining the production and distribution processes while leveraging personalized medicine advancements, such as biomarkers and genomics, can tailor vaccines to individual patients’ needs. Addressing immune evasion and the suppressive TME demands a deeper understanding of tumor immune escape mechanisms, potentially leading to novel strategies for circumventing immune suppression and improving cell-based vaccine efficacy. Additionally, optimizing peptide vaccines entails broadening the range of tumor antigens considered and exploring heteroclitic peptides to expand their applicability across various HLA types. Combining vaccines with immune checkpoint inhibitors or other immunomodulatory agents could synergistically enhance vaccine responses. For iPSC-based vaccines, rigorous preclinical safety assessments and refined protocols for generating iPSCs are paramount for safe and reliable clinical translation. Furthermore, exosome-based vaccines could benefit from improved production methods and enhanced loading techniques to ensure potent and consistent vaccines with sufficient tumor antigens. Understanding the interactions between exosomes and the immune system will provide insights into their immunomodulatory effects and safety profiles. Conducting well-designed clinical trials with long-term follow-ups is essential to assess the efficacy, safety, and durability of cancer vaccines across diverse patient populations. By collectively pursuing these avenues of research, we can overcome current limitations, ushering in an era of more effective and personalized cancer immunotherapies that hold the promise of controlling and defeating cancer.

To overcome these challenges, ongoing research is focused on TME profiling, molecular pathway mapping, and an improved understanding of TME-mediated patterns. The aim is to optimize the development of personalized cancer vaccines and mitigate the potential risks. By gaining deeper insights into the intricate dynamics of the TME, we can refine the design of these vaccines and strive for curative outcomes, particularly for patients with advanced metastatic diseases. While TME heterogeneity, the presence of immunosuppressive cells, and tumor escape mechanisms pose significant hurdles, combination therapies that incorporate diverse vaccine types, adjuvants, and chemotherapeutic/radiotherapeutic strategies are showing promise in enhancing immune activation and improving treatment outcomes. Although the success rates of current cancer vaccines vary, continued TME profiling and molecular pathway mapping offer valuable opportunities to advance our understanding of TME-mediated patterns.

## Figures and Tables

**Table 1:** Stimulus, T cell Response and Accessory Cells utilized in vaccine types discussed in this article.

Vaccine Type	Stimulus	T Cell Response	Accessory Cells
[22,23] Cell-based vaccines	Activation of innate immune cells	CD8+ CTLs and CD4+ Th cells	NK cells, macrophages, DCs
[24] Induced pluripotent stem cell-based vaccine	Recognition of vaccine components by immune cells	CD8+ CTLs and CD4+ Th cells	NK cells, macrophages, DCs
[19] In situ cancer vaccine	Activation of immune cells in TME	CD8+ CTLs and CD4+ Th cells	DCs, macrophages, neutrophils, NK cells
[22,25] Microbial Vector Vaccines	Direct activation of immune response	CD8+ CTLs and CD4+ Th cells	Pro-inflammatory cytokines DCs, macrophages, NK cells
[22,26,27] Peptide Vaccine	Recognition of peptide antigens	CD8+ CTLs and CD4+ Th cells	APCs
[22,28,29] Nucleic acid-based vaccine	Activation of innate immune cells	CD8+ CTLs and CD4+ Th cells	APCs
[23,30] Exosome-based vaccine	Activation of dendritic cells	CD8+ CTLs and CD4+ Th cells	Dendritic cells
